# ID1 mediates resistance to osimertinib in EGFR T790M-positive non-small cell lung cancer through epithelial–mesenchymal transition

**DOI:** 10.1186/s12890-021-01540-4

**Published:** 2021-05-15

**Authors:** Kejun Liu, Xianwen Chen, Ligang Wu, Shiyuan Chen, Nianxin Fang, Limin Cai, Jun Jia

**Affiliations:** 1 Dongguan Institute of Clinical Cancer Research, Affiliated Dongguan People’s Hospital, Southern Medical University, Dongguan, China; 2 Department of Pathology, Gaozhou People’s Hospital, Maoming, China; 3 Dongguan Institute of Respiratory and Critical Care Medicine, Affiliated Dongguan People’s Hospital, Southern Medical University, Dongguan, China

**Keywords:** ID1, Osimertinib, Drug resistance, EGFR T790M, Non-small-cell lung cancer (NSCLC), Epithelial–mesenchymal transition (EMT)

## Abstract

**Background:**

ID1 is associated with resistance to the first generation of EGFR tyrosine kinase inhibitors (EGFR-TKIs) in non-small cell lung cancer (NSCLC). However, the effect of ID1 expression on osimertinib resistance in EGFR T790M-positive NSCLC is not clear.

**Methods:**

We established a drug-resistant cell line, H1975/OR, from the osimertinib-sensitive cell line H1975. Alterations in ID1 protein expression and Epithelial–mesenchymal transition (EMT)-related proteins were detected with western blot analysis. RT-PCR was used to evaluate the differences of gene mRNA levels. ID1 silencing and overexpression were used to investigate the effects of related gene on osimertinib resistance. Cell Counting Kit-8 (CCK8) was used to assess the proliferation rate in cells with altered of ID1 expression. Transwell assay was used to evaluate the invasion ability of different cells. The effects on the cell cycle and apoptosis were also compared using flow cytometry.

**Results:**

In our study, we found that in osimertinib-resistant NSCLC cells, the expression level of the EMT-related protein E-cadherin was lower than that of sensitive cells, while the expression level of ID1 and vimentin were higher than those of sensitive cells. ID1 expression levels was closely related to E-cadherin and vimentin in both osimertinib-sensitive and resistant cells. Alteration of ID1 expression in H1975/OR cells could change the expression of E-cadherin. Downregulating ID1 expression in H1975/OR cells could inhibit cell proliferation, reduce cell invasion, promote cell apoptosis and arrested the cell cycle in the G1/G0 stage phase. Our study suggests that ID1 may induce EMT in EGFR T790M-positive NSCLC, which mediates drug resistance of osimertinib.

**Conclusions:**

Our study revealed the mechanism of ID1 mediated resistance to osimertinib in EGFR T790M-positive NSCLC through EMT, which may provide new ideas and methods for the treatment of EGFR mutated NSCLC after osimertinib resistance.

**Supplementary Information:**

The online version contains supplementary material available at 10.1186/s12890-021-01540-4.

## Background

Lung cancer, one of the most common malignant tumors and also the primary cause of death in cancer patients worldwide, is classified into small-cell lung cancer and non-small cell lung cancer (NSCLC); the latter of which accounts for appropriately 80% of all cases of lung cancer cases [1, 2]. For patients with advanced NSCLC harboring epidermal growth factor receptor (EGFR) gene mutations, which accounts for 30–50% of NSCLC cases in East Asia, the first generation of EGFR tyrosine kinase inhibitors (EGFR-TKIs) such as gefitinib and erlotinib have superior therapeutic efficacy compared to traditional platinum-based chemotherapy [3, 4] and the median progression-free survival (PFS) is 9–13 months. However, these patients will eventually suffered secondary drug resistance during the targeted therapy and most of them may harbor the T790M mutation in exon 20 of the EGFR gene [5]. For patients with the EGFR T790M mutation, osimertinib still shows favorable therapeutic effect [6]. The median PFS of patients with NSCLC after failure of the first generation of EGFR-TKIs treatment is significantly higher than patients receiving standard platinum-based chemotherapy [7]. However, osimertinib may also develop drug resistance.

Epithelial–mesenchymal transition (EMT) may be one of the mechanisms that mediates osimertinib resistance. Previous studies have shown that resistance to gemcitabine in EMT producing pancreatic cancer cell lines was significantly increased [8]. Similarly, EMT has been observed in fluorouracil resistant colon cancer cell lines [9], tamoxifen resistant breast cancer cell lines [10], cisplatin resistant cervical cancer cell lines [11], and gefitinib resistant lung cancer cell lines [12]. Rastogi I et al. reported that in EGFR-TKIs resistant cell lines, ZEB1 was overexpressed and E-cadherin expression was inhibited, thus inducing EMT in lung cancer cells [13]. After down-regulating ZEB1 expression with miR-200a or β-catenin siRNA, EGFR-TKIs sensitivity could be restored. Recently, it has been found that the Hedgehog signaling pathway is abnormally activated in EGFR-TKIs resistant cells [14]. Blocking the Hedgehog signaling pathway with SANT-1 could restore the expression of E-cadherin and increase the sensitivity of cells to EGFR-TKIs. In EGFR-TKIs sensitive cells, up-regulation of the Hedgehog signaling pathway could inhibit the expression of E-cadherin and increase the expression of Snail and ABCG2, leading to drug resistance. Therefore, these studies suggest that EMT plays an important role in mediating EGFR-TKIs resistance in lung cancer.

The process of EMT is very complex, and involves multiple signaling pathways. EMT can be induced by the NF-κB/snail/PTEN loop, Notch-2 and MAPK/mitochondria. It has been shown that when EMT occurred in tumor cells, epithelial markers such as E-cadherin were lowly expressed while mesothelial markers such as vimentin and N-cadherin were highly expressed [15]. Therefore, inhibition of E-cadherin expression can induce EMT. The exogenous signal that promote tumor growth can inhibit the expression of E-cadherin and mediate EMT by forming complex with transcription factors such as Snail, Slug, ZEB1, SIP1 and Twist and binding to the promoter of the CDH1 gene [5]. In addition, many microRNAs can regulate the expression of E-cadherin and induce the production of EMT in tumor cells by regulating the transcription repressors ZEB1 and SIP1.

ID1 belongs to the basic helix-loop-helix (bHLH) transcription factor family, and is hardly expressed in adult tissues and cells [16]. However, it has been reported that ID1 is highly expressed in a variety of tumors and is involved in cell proliferation, cell cycle progression, differentiation inhibition and genomic instability. Studies have shown that ID1 can promote the growth of esophageal cancer cells through PI3K/Akt/NF-κB signaling pathway [17]. Inhibition of ID1 can lead to the inactivation of NF-κB, thus promoting the apoptosis of colorectal cancer cells [18]. Previous studies have also reported that ID1 is highly expressed in lung cancer cells. ID1 is associated with EGFR-TKIs resistance in lung cancer. The higher the expression of ID1, the stronger the resistance of cells to EGFR-TKIs [19]. Furthermore, the higher the expression of ID1, the worse the prognosis of NSCLC. It is suggested that ID1 may inhibit the PI3K/AKT signaling pathway through negative feedback. As NF-κB is the downstream signal pathway of PI3K/Akt, the increase of ID1 expression may lead to EMT through the NF-κB/Snail/PTEN loop as illustrated above.

The aim of our study was to reveal the mechanism of ID1 mediated resistance to osimertinib in EGFR T790M-positive NSCLC through EMT, and to provide new ideas and methods for the clinical treatment of EGFR mutated NSCLC after osimertinib resistance.

## Methods

### Chemicals and reagents

Osimertinib was obtained from Selleck Chemicals (Houston, TX, USA). RPMI-1640 was product of HyClone (Logan, UT, USA) and Dulbecco’s modified Eagle’s medium (DMEM) and fetal bovine serum (FBS) were products of Gibco BRL (Grand Island, NY, USA). Trizol was purchased from Takara Biomedical Technology (Shiga, Japan). Cell Counting Kit-8 (CCK-8) was obtained from Dojindo (Kumamoto, Japan). Monoclonal antibodies against ID1 were products of Santa Cruz Biotechnology (Santa Cruz, CA, USA). Glyceraldehyde-3-phosphate de hydrogenase (GAPDH), E-cadherin and vimentin antibodies were purchased from Abcam (Cambridge, MA, USA).

### Cell culture and transfection

The EGFR mutant human lung adenocarcinoma cell lines, H1975, were purchased from the Type Culture Collection of the Chinese Academy of Sciences, Shanghai, China. H1975/OR was obtained by gradually increasing the concentration of osimertinib into H1975 cell culture medium. Cells were cultured in RPMI-1640 medium supplemented with 10% FBS (Gibco, Grand Island, NY, USA) at 37 °C in a humidified incubator with 5% CO2. All cells were grown in culture medium that is free of drugs for more than 14 days before assay. Cells were seeded into 96-well Falcon plates with 1.0 × 10^5^ cells per well. H1975/OR cells were divided into 6 groups: the untreated group, empty vector group, interference control group, interference group, overexpression group, both interference and overexpression group. Transient transfections of H1975/OR cells were performed with the lipofectamine 2000 according to the manufacturer's instructions when the cell confluence was approximately 90%. Cells were cultivated for further 48 h prior to the following experiments.

### Cell proliferation assay

Cells were seeded at a density of 1.0 × 10^4^ cells/well in a 96-well plate for 48 and 72 h. The CCK-8 solution was added to each well prior to the endpoint of incubation. The CCK-8 reagent was added into each well at a 1:10 (v/v) dilution per 100 µL and incubated for 2 h at 37 °C. We quantified the results spectrophotometrically at a wavelength of 450 nm.

### Transwell assay

At 48 h post-transfection, cells (5 × 10^4^) were cultured in medium without serum. A Transwell assay (pore size, 8 µm; Corning, Inc.) was performed to detect cell invasion. Cells (2 × 10^5^ cells/ml) were plated into the upper chamber with medium containing 1% FBS, which was pre-coated with Matrigel^®^ at 37 °C for 30 min. Medium supplemented with 10% FBS (500 µl) was plated in the lower chambers. Following incubation for 48 h at 37 °C, cells on the upper chamber surface were removed. Invading cells were fixed with 50% methanol for 30 min at 4 °C and stained with 0.1% crystal violet for 30 min at room temperature. Stained cells were visualized under a light microscope and the number of invading cells was calculated to determine the relative invasion rate.

### Western blot analysis

Cells were lysed after washing 2 times with ice-cold phosphate-buffered saline (PBS). The protein concentration was quantified using the Bradford method. Equal amounts of protein were resolved by sodium dodecyl sulfate polyacrylamide gel electrophoresis (SDS-PAGE) and transferred onto nitrocellulose membranes. Membranes were blocked with 5% fat-free milk combined with Tris-buffered saline for one hour at room temperature and then incubated with the appropriate primary antibody and horseradish peroxidase conjugated secondary antibody. Chemiluminescence was used to detect the proteins. Protein expression was quantified using Image-Pro Plus software (version 6.0; Media Cybernetics, Inc.) with GAPDH as the loading control.

### Reverse transcription PCR

ID1 expression was assayed as described. Total RNA was isolated using the Trizol reagent RNA extraction kit (Takara Bio Inc., Shiga, Japan) according to the manufacturer’s protocol. Then, cDNA was synthesized using the BestarTM qPCR RT kit (DBI Bioscience), following the manufacturer’s instructions. Quantitative real-time PCR analysis was performed with the BestarTM qPCR MasterMix (DBI Bioscience). Each sample was run in triplicate for each gene. Transcript levels were normalized to the housekeeping gene phosphoglycerate kinase (PGK) and analyzed by the relative quantification 2−ΔΔCt method. The PCR primers were as follows: GAPDH: forward: 5′-TGTTCGTCATGGGTGTGAAC-3′, reverse:5′-ATGGCATGGACTGTGGTCAT-3′, Vimentin:forward:5′-AGTCCACTGAGTACCGGAGAC-3′, reverse:5′-CATTTCACGCATCTGGCGTTC-3′, E-cadherin: forward:5′-ATTTTTCCCTCGACACCCGAT-3′, reverse:5′-TCCCAGGCGTAGACCAAGA-3′, ID1:forward:5′-CTGCTCTACGACATGAACGG-3′, reverse:5′-GAAGGTCCCTGATGTAGTCGAT-3′. The products were resolved using gel electrophoresis (1.5% agarose gel).

### Flow cytometry analysis of apoptosis

Cells were incubated with 0.25% trypsin. Then, the cells were observed under the microscope. When the cytoplasm was retracted and the cells were detached, the trypsin was removed. Then, 4 ml of complete culture medium was added to stop digestion and single cell suspension was made. The single cell suspensions were transferred to flow tubes, washed with PBS twice and centrifuged at 1000 rpm for 5 min. The supernatants were discarded, then 200 μl annexin V-FITC/propidium iodide (PI) staining solution was used to resuspend the cells. The cells were incubated in the dark for 15 min and staining was detected using a flow cytometer.

### Flow cytometry analysis of the cell cycle

To determine the effects of ID1 on the cell cycle, 2 × 10^4^ cells/well were seeded in 6-well plates and incubated for 24 h. Cells were removed and single cell suspensions were made. The single cell suspensions were then transferred to flow tubes, washed with PBS twice and centrifuged at 1000 rpm for 5 min. The supernatant was completely removed as much as possible, and 1 ml 0.25% trypsin was added. Cells were resuspended with 70% ethanol precooled at − 20 °C. The samples were stored at − 20 °C. The samples were centrifuged at 1000 rpm for 5 min each time, washed with PBS for 1–2 times and centrifuged again at 1000 rpm for 5 min. Next, 300 μl annexin V-FITC/PI staining solution was added to cells and incubated for 15 min in the dark, then the staining was detected and analyzed using a flow cytometer.

### Statistical analysis

Statistical analyses were performed using SPSS software (version 22.0; IBM Corp). Data are presented as the mean ± standard deviation. All experiments were repeated at least three times and the differences were analyzed by using the Student’s t-test. The significance was determined at P < 0.05.

## Results

### Generation of the osimertinib-resistant cell line H1975/OR

We selected the NSCLC cell line H1975 in our study. H1975 cells harbor the EGFR T790M mutation and are therefore sensitive to osimertinib. Its corresponding drug resistant cell line H1975/OR was induced by osimertinib. In detail, H1975 cells were exposed to the culture medium with increasing concentration of osimertinib. Once the resistant cell line was confirmed, H1975/OR cells were screened by gene sequencing. We found that there were no secondary gene mutations occurred, such as C797S, amplifification of MET or loss of EGFR T790M. These cells were cultured in RPMI-1640 medium containing 10% FBS at 37 °C with 5% CO_2_.

### Different expression of ID1 and EMT related protein

**Fig. 1:**
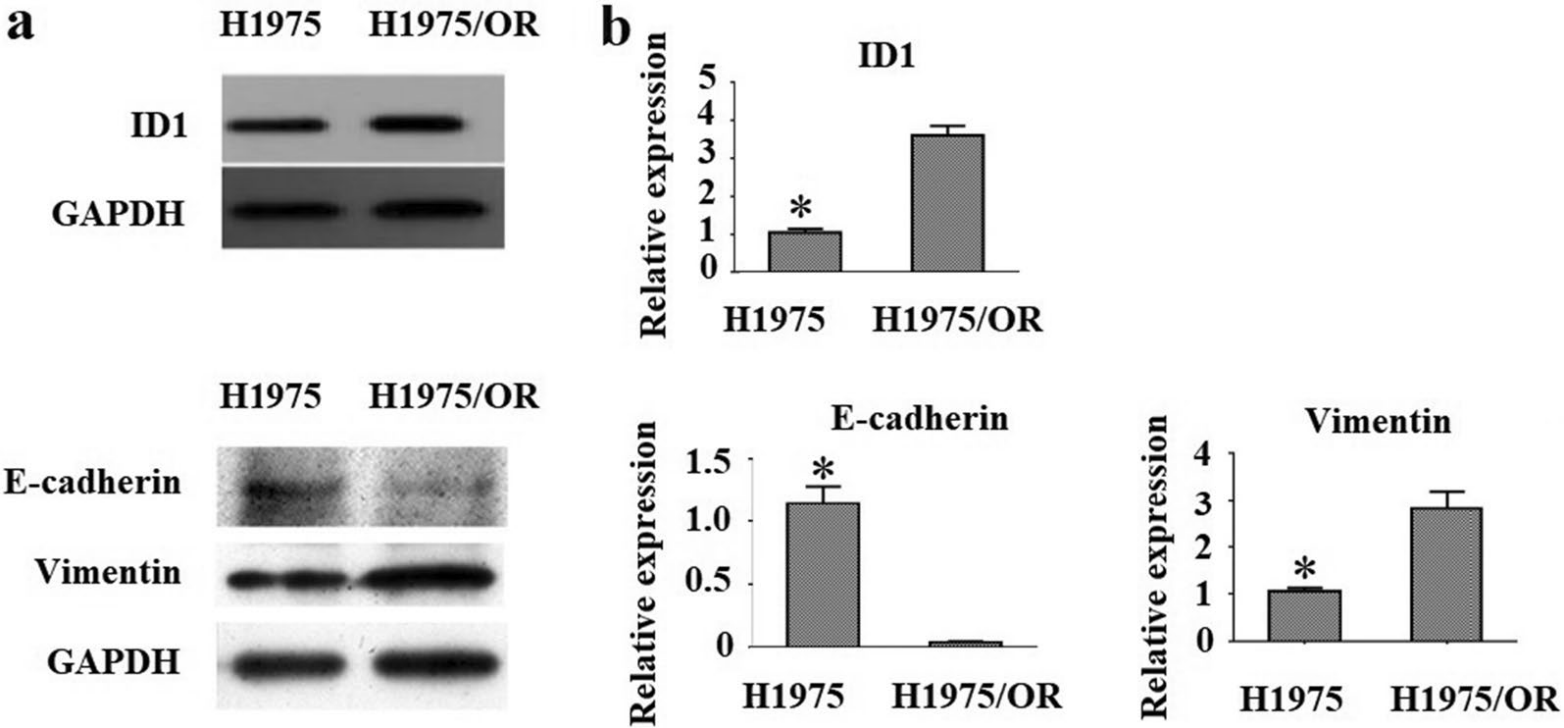
Western blot analysis and Reverse transcription PCR of ID1 and EMT related protein. **a** Western blot and **b** RT-PCR were used to detect the expression of ID1, E-cadherin, and vimentin in H1975 and H1975/OR cells. The mRNA and protein expression level of E-cadherin in H1975/OR cells was lower than that in H1975 cells, while the expression levels of ID1 and vimentin in H1975/OR cells were higher than those in H1975 cells. Representative results are shown from at least 3 independent experiments.*P < 0.05 versus H1975/OR

In the study, we detected the expressions of ID1 and EMT-related protein at the mRNA and protein level using reverse transcription-PCR (RT-PCR) and western blot analysis. At the protein level, ID1 and vimentin appeared to be up-regulated in the H1975/OR cell line as compared to the osimertinib-sensitive cell line H1975, whereas the expression of E-cadherin was significantly decreased in H1975/OR cells (Fig. 1 and Additional files 1 and 2). The mRNA levels of ID1, vimentin and E-cadherin were also changed accordingly. These results suggested that ID1 expression level was closely related to E-cadherin and vimentin both in both osimertinib-sensitive and resistant cells. Therefore, overexpression of ID1 may be one of the mechanisms of osimertinib resistance to EGFR T790M-positive NSCLC cells through EMT.

### Impact of ID1 on the expression of EMT-related proteins

**Fig. 2:**
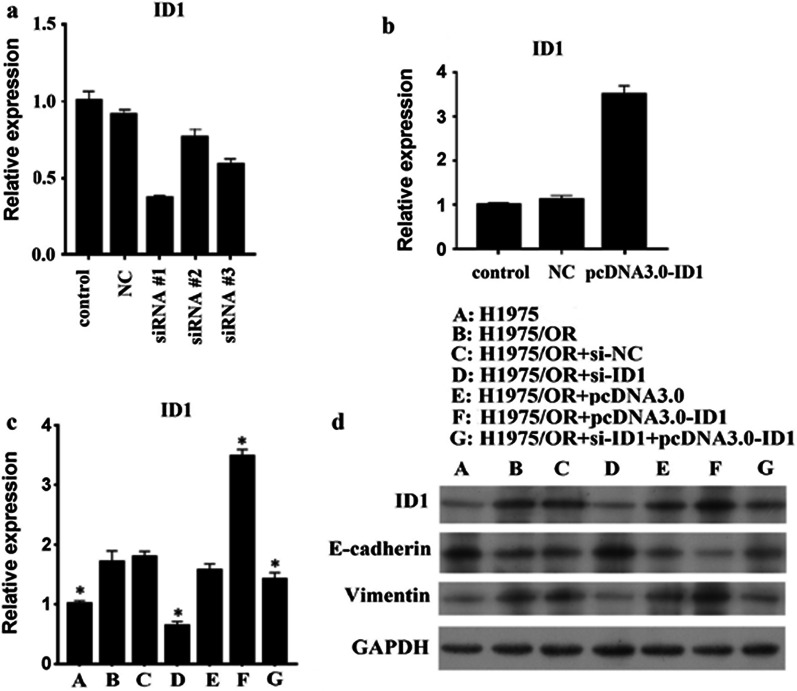
Ralationship between different ID1 expression and EMT related protein. **a** Gene silencing using siRNA targeting ID1 and **b** overexpression of ID1 by cloning the gene into the pcDNA3.0 vector. **c** and **d** RT-PCR and western blot were used to confirmed the effects of ID1 silencing and overexpression on osimertinib-resistant cells. Results showed that the expression of ID1 was closely related to E-cadherin and vimentin. Up- and down-regulation of ID1 expression in H1975/OR cells inhibited and promoted the expression of E-cadherin, respectively. Each point represents the mean ± SD from 3 independent experiments.*P < 0.05 versus H1975/OR

To further understand and investigate the overexpression of ID1 in relation to osimertinib resistance in H1957/OR cells, we performed gene silencing using siRNA targeting ID1 and overexpression by cloning the ID1 gene into the pcDNA3.0 vector. RT-PCR and western blot confirmed the effect of ID1 silencing and overexpression on drug-resistant cells. Down-regulation of ID1 expression in H1975/OR cells promoted the expression of E-cadherin and decreased the expression of vimentin (Fig. 2 and Additional file 3). Conversely, in the negative control cell line, both the expression of E-cadherin and vimentin did not changed significantly as compared to that in the H1957/OR cell line. Our study indicated that overexpression of ID1 could induce EMT in EGFR T790M-positive NSCLC cells, which may mediate drug resistance to osimertinib.

### Impact of ID1 on the proliferation and invasion of osimertinib-sensitive and resistant cells

**Fig. 3:**
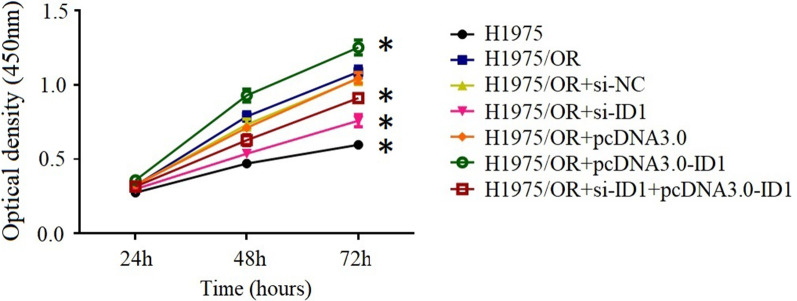
Cell proliferation assay of osimertinib-sensitive and resistant cells. Results showed that cell proliferation rate was lowest in H1975 cells. Down-regulating ID1 could decrease proliferation rate in H1975/OR + si-ID1 cells, while overexpression of ID1 could further increase cell proliferation in H1975/OR + pcDNA3.0-ID1 cells. The values represent mean ± SD from 3 independent experiments. *P < 0.05 versus H1975/OR

**Fig. 4:**
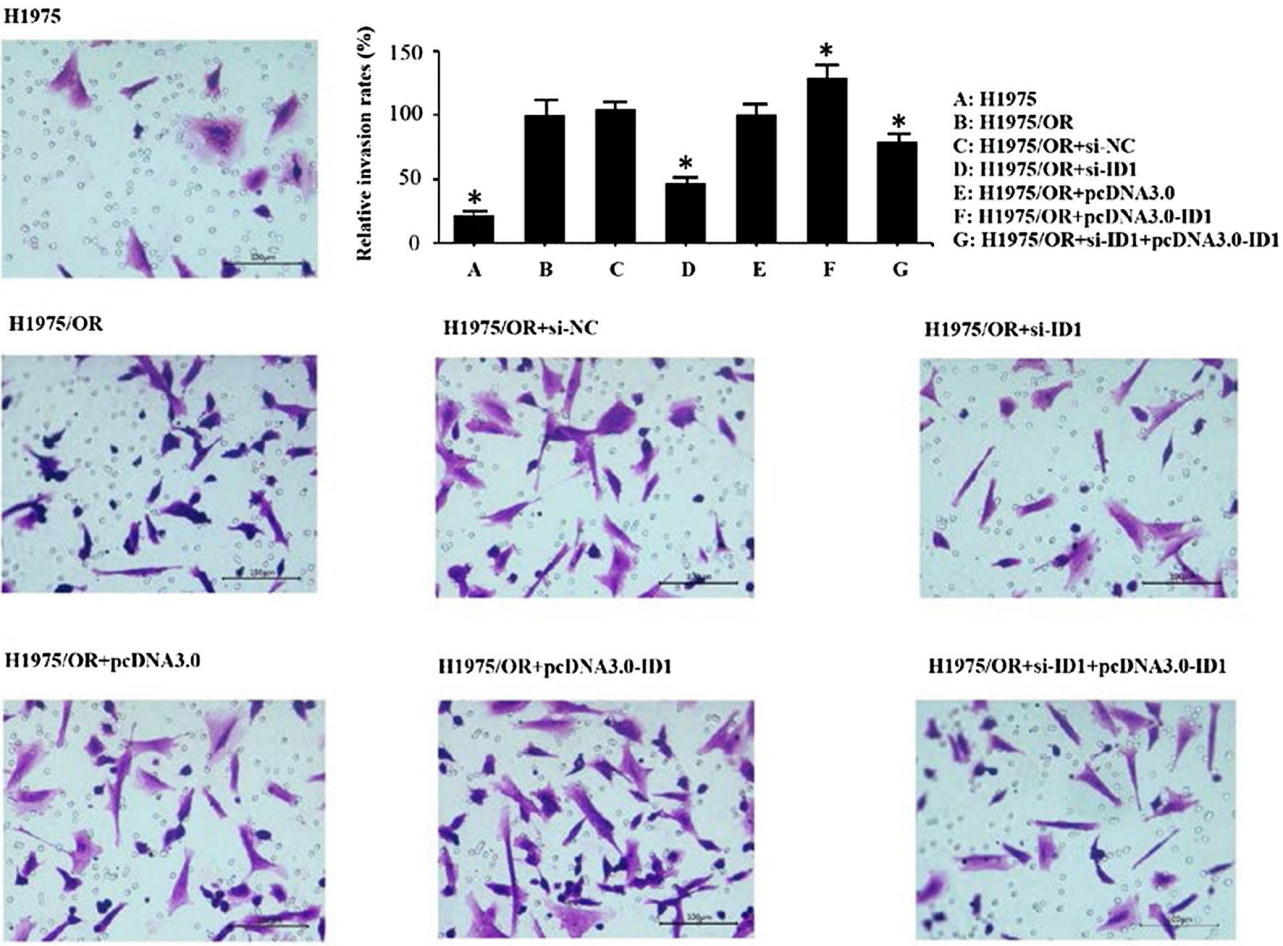
Transwell assay of osimertinib-sensitive and resistant cells. Results showed that cell invasion was lowest in H1975 cells. Down-regulating ID1 could decrease cell invasion in H1975/OR + si-ID1 cells, while overexpression of ID1 could further enhance cell invasion in H1975/OR + pcDNA3.0-ID1 cells. The values represent mean ± SD from 3 independent experiments. *P < 0.05 versus H1975/OR

We then investigate whether alteration in ID1 expression could change cell proliferation of H1975/OR cells. As is shown in Figs. 3 and 4, we observed that downregulating ID1 could inhibit cell proliferation and reduce cell invasion, while overexpression of ID1 could further increase cell proliferation and enhance cell invasion. In control cells, the cell biological behaviors did not change significantly as compared to that of H1975/OR cells.

### Impact of ID1 on apoptosis and the cell cycle of osimertinib-sensitive and resistant cells

**Fig. 5:**
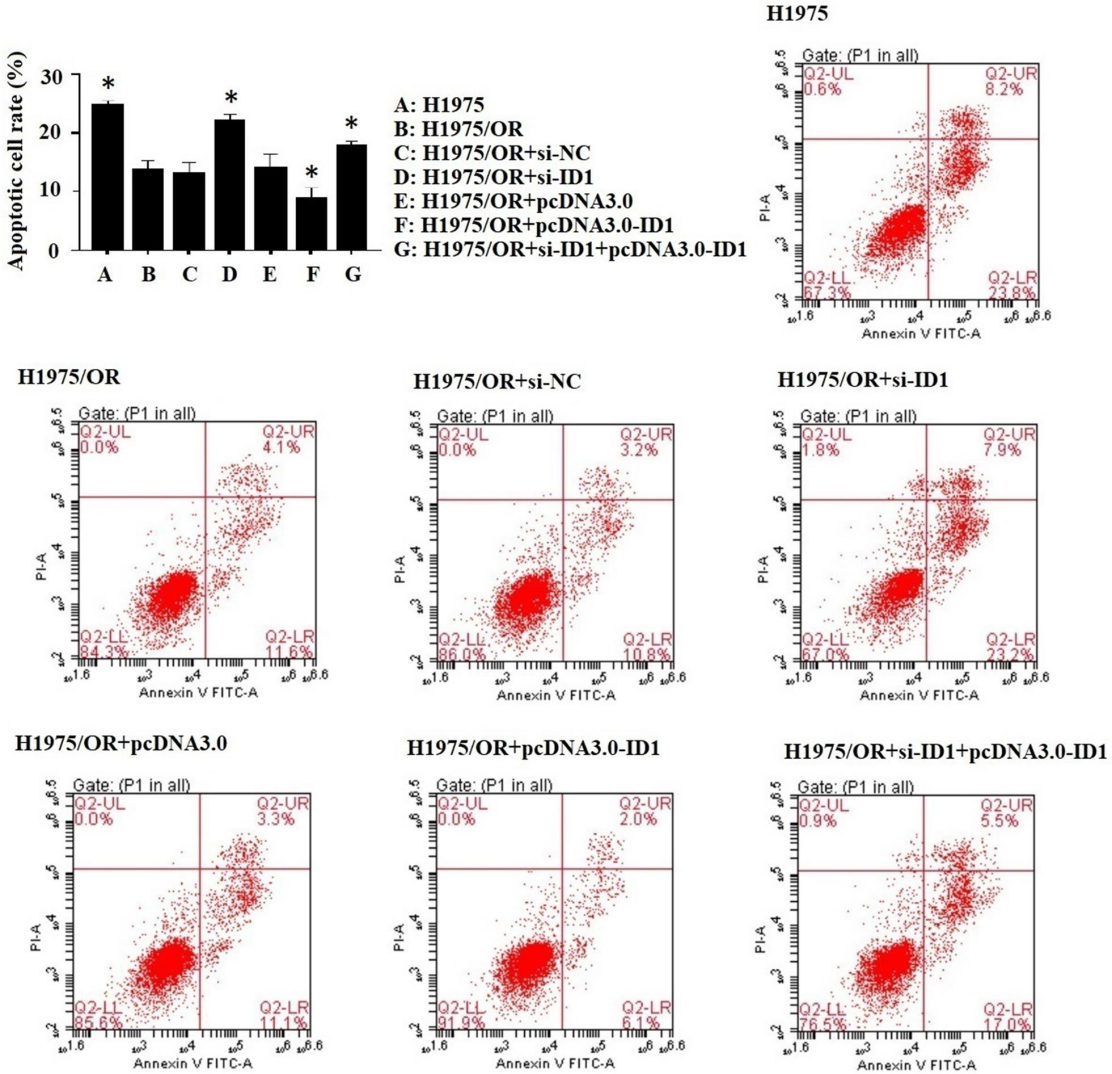
Flow cytometry analysis of apoptosis in osimertinib-sensitive and resistant cells. The apoptosis rate in H1975/OR cells was significantly decreased compared with that in osimertinib-sensitive cells (13.63 ± 1.72% versus 24.7 ± 0.78%). In H1975/OR + si-ID1 cells, the apoptosis rate was increased compared to H1975/OR cells (22.07 ± 1.03% versus 13.63 ± 1.72%), whereas in ID1 overexpressed H1975/OR + pcDNA3.0-ID1 cells, the apoptosis rate was further decreased (13.63 ± 1.72% versus 8.73 ± 1.94%). Representative results are shown from at least 3 independent experiments. *P < 0.05 versus H1975/OR

In our study, we found that alteration of ID1 expression in H1975/OR cells could change apoptosis induced by osimertinib. The apoptosis rate in H1975/OR cells was significantly decreased compared with that in osimertinib-sensitive cells (13.63 ± 1.72% versus 24.7 ± 0.78%). In H1975/OR + si-ID1 cells, the apoptosis rate was increased compared to H1975/OR cells (22.07 ± 1.03% versus 13.63 ± 1.72%), whereas in ID1 overexpressed H1975/OR + pcDNA3.0-ID1 cells, the apoptosis rate was further decreased (13.63 ± 1.72% versus 8.73 ± 1.94%) (Fig. 5). This result was in accordance with that of the sensitive cells, which indicated that by inhibiting the expression of ID1, the drug resistance of osimertinib could be reversed in H1975/OR cells. To confirm this phenomenon, we checked the result in the negative control group. The rate of cell apoptosis did not change significantly compared to that in H1975/OR cells.

**Fig. 6:**
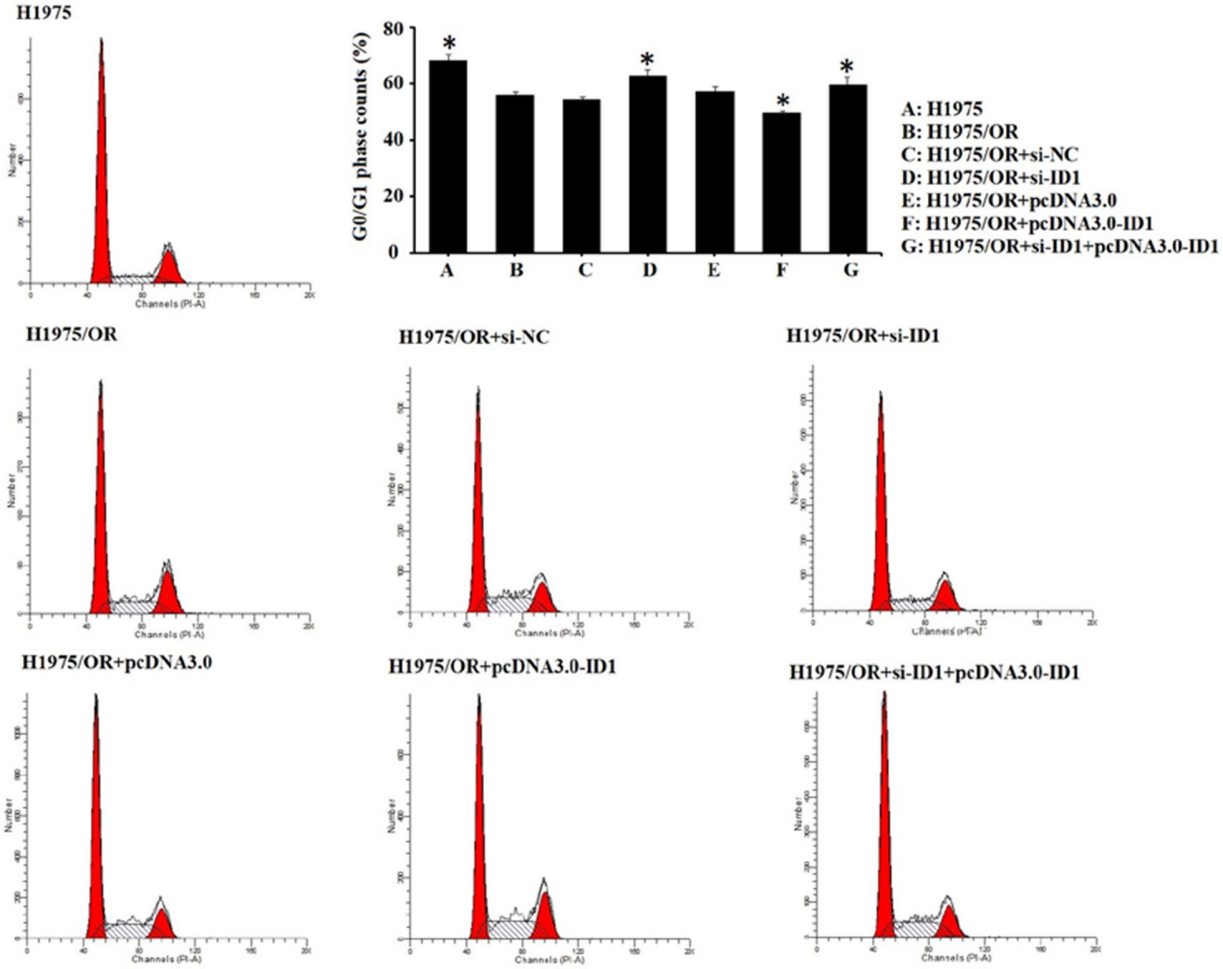
Flow cytometry analysis of the cell cycle of osimertinib-sensitive and resistant cells. Compared with H1975/OR cells, cells were arrested in the G1/G0 phase in H1975 cells (56.15 ± 0.77% versus 68.29 ± 1.24%). By down-regulating ID1, this result was reversed in H1975/OR + si-ID1 cells (56.15 ± 0.77% versus 63.05 ± 0.82%), while in ID1 overexpressed H1975/OR + pcDNA3.0-ID1 cells, the effect on cell cycle was further aggravated (56.15 ± 0.77% versus 49.96 ± 0.44%). Representative results are shown from at least 3 independent experiments.*P < 0.05 versus H1975/OR

We conducted further experiment to investigate the impact of ID1 on the cell cycle of osimertinib-sensitive and resistant cells. The ratio of different cell cycle phases in H1975/OR cells is as following: the G1/G0 phase (56.15 ± 0.77%), the S phase (25.59 ± 1.47%), the G2/M phase (18.26 ± 2.22%). As compared to H1975/OR cells, cells were arrested in the G1/G0 phase in H1975 cells (56.15 ± 0.77% versus 68.29 ± 1.24%). By down-regulating ID1, this result was reversed in H1975/OR + si-ID1 cells (56.15 ± 0.77% versus 63.05 ± 0.82%), while in ID1 overexpressed H1975/OR + pcDNA3.0-ID1 cells, the effect on cell cycle was further aggravated (56.15 ± 0.77% versus 49.96 ± 0.44%) (Fig. 6). Moreover, our results indicated that ID1 overexpression significantly reduced the number of cells in G1/G0 phase compared with the pc-control group. By contrast, ID1 knockdown increased the number of cells in G1/G0 phase compared with the si-control group, which was similar to that in the osimertinib-sensitive H1975 cells.

## Discussion

Currently, first-line treatment for patients with EGFR mutated advanced NSCLC includes single targeted agents such as osimertinib, gefitinib and erlotinib. For lung cancer patients with EGFR T790M mutation, osimertinib as first-line treatment or as subsequent treatment after initial targeted therapy should be given priority [7]. However, osimertinib may still face the problem of drug resistance after a period of therapeutic time. The mechanism of osimertinib resistance is very complex. The most important mechanism is the C797S mutation of the EGFR gene [20]. The C797S mutation is located in the EGFR tyrosine kinase region, which can inhibit the effect of osimertinib and mediate the drug resistance of lung cancer cells. For the C797S mutation, EAI045 has been developed to overcome osimertinib resistance [21].

For patients without the C797S mutation, it has been reported that HER2 or c-Met amplification pathways are involved in osimertinib resistance [22]. Abnormal activation of HER2 can activate downstream pathways such as PI3K/Akt and MEK/MAPK through the formation of homologous or heterologous dimers, which leads to abnormal proliferation of tumor cells and the production of osimertinib resistance. Additionally, c-Met amplification bypasses EGFR activation of the downstream PI3K/Akt-mediated signaling pathway, leading to osimertinib resistance. The activation of Ras, a downstream signaling pathway, can also lead to osimertinib resistance [23]. In addition to these processes, there are still many unknown mechanisms of osimertinib resistance, especially in the front-line settings. Therefore, investigating the resistance mechanism of osimertinib and overcoming the resistance problem of the third generation EGFR-TKIs have become one of the research focuses for NSCLC.

Till date, several resistance mechanisms have been described in previous studies such as the FLAURA and AURA3 trials. However, it was still unclear whether other mechanisms of acquired resistance to osimertinib existed when used as front line treatment. As were shown in previous studies, EMT can be resistant to traditional anticancer drugs and EGFR-TKIs. EMT can promote the growth, migration and metastasis of tumor cells. It has been found that EMT tumor cells have the ability for self-renewal, unlimited proliferation and anti-apoptosis, and highly express CD133, CD44 + and ABCG2. These characteristics are very similar to those of cancer stem cells, which may be the potential mechanism of osimertinib resistance [24].

In our study, we found that in osimertinib-resistant NSCLC cells, the expression level of the EMT-related protein E-cadherin was lower than that in sensitive cells, while the expression level of ID1 and vimentin were higher than those in sensitive cells. Furthermore, ID1 expression level was closely related to E-cadherin and vimentin both in both osimertinib-sensitive and resistant cells. Alteration of ID1 expression in H1975/OR cells could change the expression of E-cadherin. Downregulating ID1 expression in H1975/OR cells could inhibit cell proliferation, reduce cell invasion, promote cell apoptosis and arrested the cell cycle in the G1/G0 stage phase. Our study indicated that ID1 could induce EMT in EGFR T790M-positive NSCLC cells, which mediates drug resistance to osimertinib. These results may compensate the pre-existing osimertinib resistance mechanisms such as C797S mutation, c-Met amplification and downstream pathways activation.

In previous studies, ID1 was shown to participate in liver metastasis of lung cancer cells through EMT and knockout of ID1 may lead to decreased expression of vimentin, TGF-β and Snail [25]. Down-regulation of ID1 expression can not only inhibit EMT formation, but can also induce tumor apoptosis. It has been reported that ID1 knockout in ovarian cancer cells significantly inhibited the growth and invasion of tumor cells and promoted the apoptosis of tumor cells [26]. In colon cancer HCT116 cells, ID1 inhibited apoptosis induced by chemotherapy drugs and ultraviolet light [27]. In small cell lung cancer, it has been reported that the high expression of ID1 can significantly inhibit the apoptosis of tumor cells [28]. Our study is therefore consistent with the results of these former studies.

In head and neck cancer cells, Snail-induced EMT enabled cancer cells to maintain their tumor stem cell-like properties, thus increasing resistance to chemotherapy and invasiveness [29]. When tumor cells produce EMT, they often secrete more cytokines such as CXCL9 and CXCL10, which can inhibit the function of NK cells, thus promoting tumor immune escape [30]. Our study found that in EGFR T790M-positive NSCLC cells, increased ID1 expression could mediate EMT induction, inhibit cell apoptosis and promote cell proliferation. Down-regulation of ID1 expression could arrest the cell cycle in the G1/G0 phase. After ID1 expression level changes, does the proportion of tumor stem cells change? At present, there is no report that ID1-mediated EMT promotes the formation of tumor stem cells to regulate the immune escape of EGFR T790M-positive lung cancer. We will therefore conduct further studies to investigated these mechanisms.

However, our study has several limitations. Firstly, the role of ID1 in osimertinib resistance was only explored in H1975 and H1975/OR cells. It is difficult to confirm whether ID1 is definitely related to the drug resistance of osimertinib in other cell lines. Therefore, further studies are warranted to include more NSCLC cell lines harboring EGFR T790M mutation. Secondly, due to a shortage in time and funding, several experiments were not investigated in the current study, such as the correlation between ID1, EMT downstream signaling pathways and immune escape after osimertinib resistance. Furthermore, our study was only conducted in vitro, and in vivo experiments in models such as the nude mouse xenograft model were not performed. Hence, the role of ID1 needs to be carefully verified in animal experiments. Lastly, our study did not involved in human samples as compared to other similar researches. As a consequence, further exploration of ID1 in EGFR T790M-positive lung cancer cells is required.

## Conclusions

In conclusion, our study revealed the mechanism of ID1 mediated resistance to osimertinib in EGFR T790M-positive NSCLC through EMT, which may provide new ideas and methods for the clinical treatment of EGFR mutated NSCLC after osimertinib resistance. Further studies are needed to elucidate the underlying mechanism of osimertinib resistance in first-line targeted therapy.

## Supplementary Information

**Additional file 1.** Western blot of ID1 expression.

**Additional file 2.** Western blot of EMT-related proteins.

**Additional file 3.** Western blot of different ID1 expression and EMT-related proteins.

## Data Availability

The datasets used and analyzed during the current study are available from the corresponding author on reasonable request.

## Abbreviations

NSCLC: Non-small cell lung cancer
EGFR: Epidermal growth factor receptor
TKIs: Tyrosine kinase inhibitors
EMT: Epithelial–mesenchymal transition
PFS: Progression-free survival
bHLH: Basic helix-loop-helix
CCK-8: Cell Counting Kit-8 assay
SDS-PAGE: Sodium dodecyl sulfate polyacrylamide gel electrophoresis
PGK: Phosphoglycerate kinase
RT-PCR: Reverse transcription-PCR
