# Supplementary figures and images for: ID1 mediates resistance to osimertinib in EGFR T790M-positive non-small cell lung cancer through epithelial–mesenchymal transition
Source: BMC Pulm Med. 2021 May 15;21:163. doi: 10.1186/s12890-021-01540-4 (PMC8126145; doi:10.1186/s12890-021-01540-4)

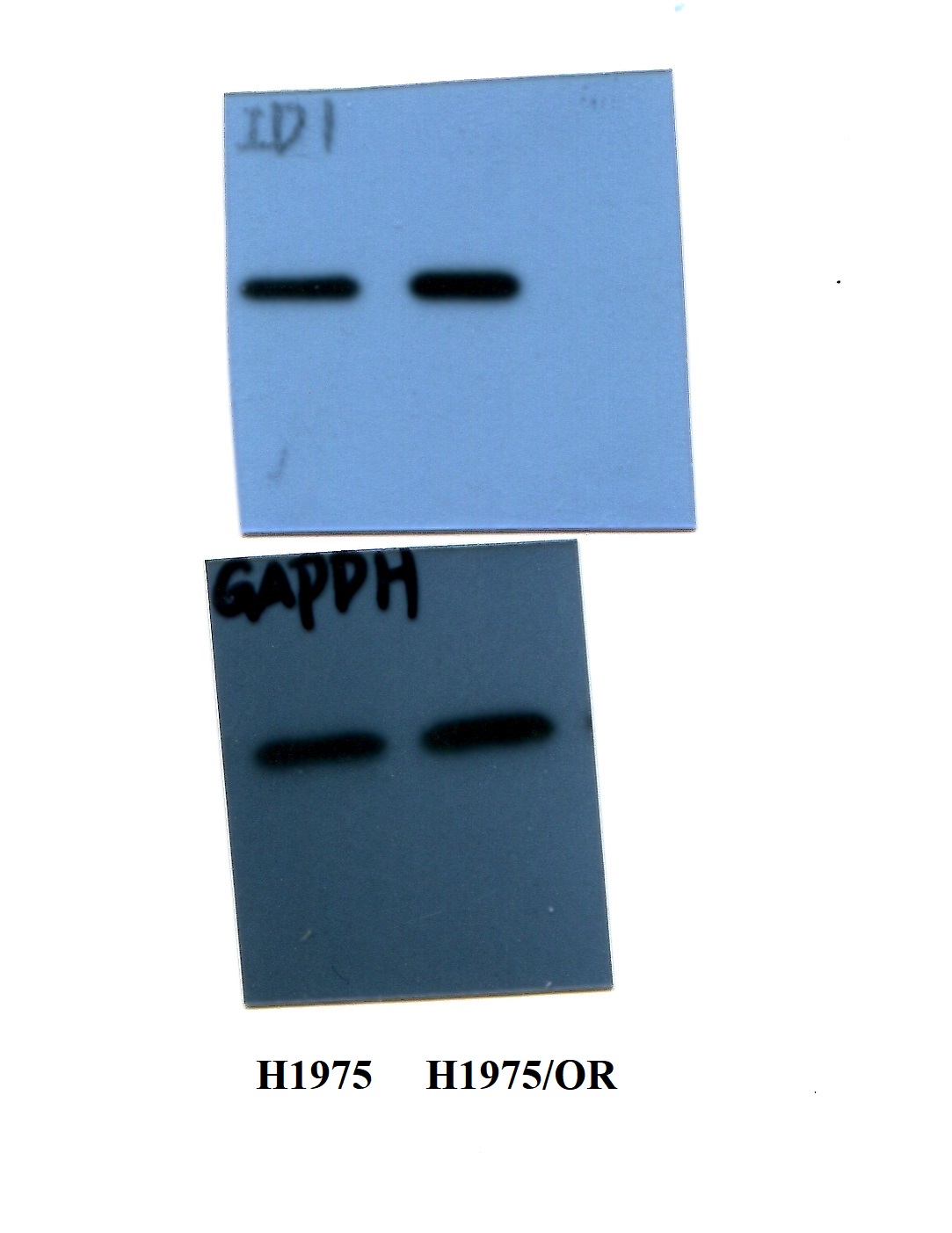

Supplement: Supplementary file 1 — Additional file 1. Western blot of ID1 expression. [file 12890_2021_1540_MOESM1_ESM.jpg]

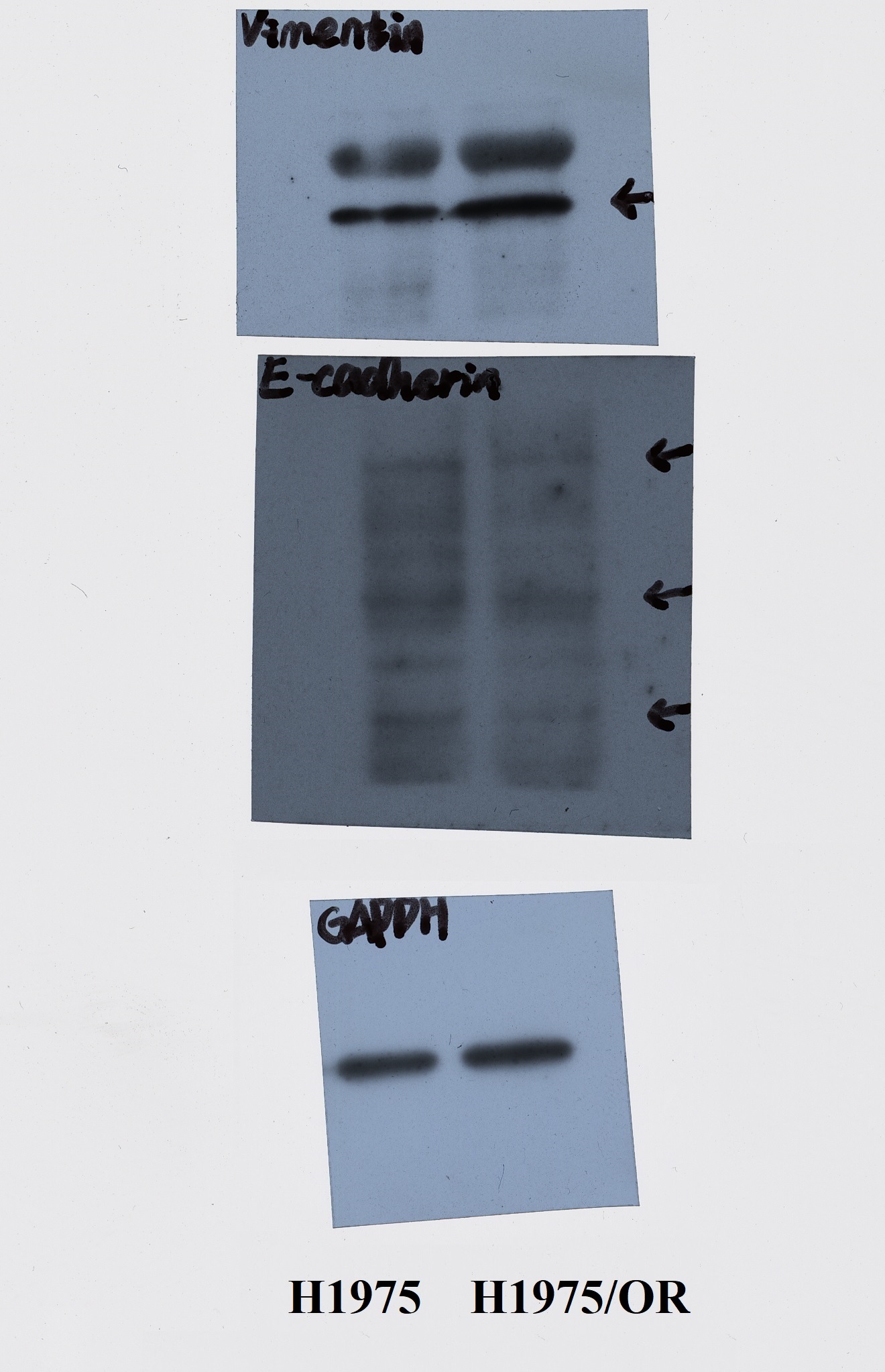

Supplement: Supplementary file 2 — Additional file 2. Western blot of EMT-related proteins. [file 12890_2021_1540_MOESM2_ESM.jpg]

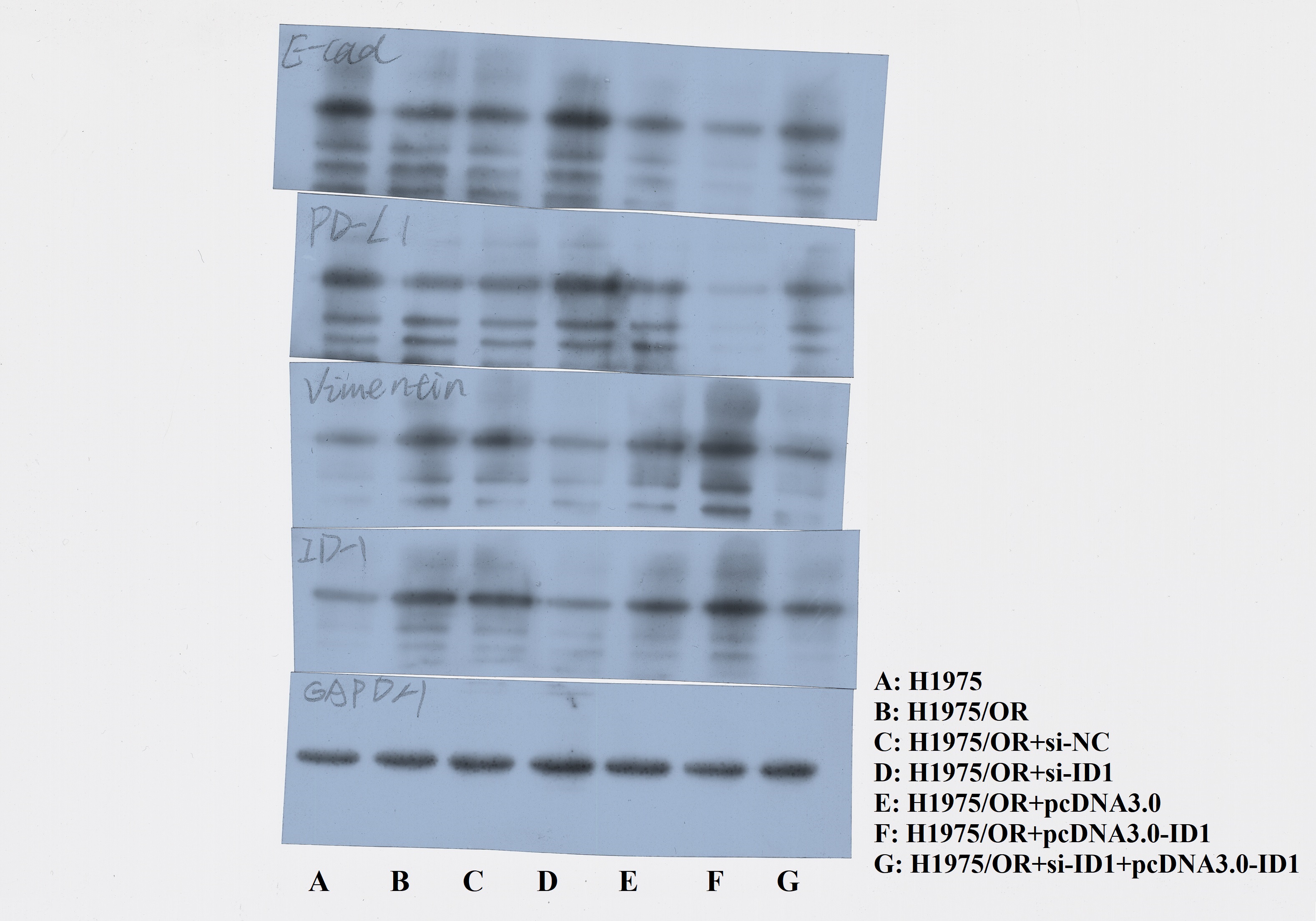

Supplement: Supplementary file 3 — Additional file 3. Western blot of different ID1 expression and EMT-related proteins. [file 12890_2021_1540_MOESM3_ESM.jpg]
